# Uptake and acceptability of health services for adolescent girls and young women provided through Safe Spaces in South Africa

**DOI:** 10.3389/fpubh.2026.1797269

**Published:** 2026-03-30

**Authors:** R. Stofile, B. Sindi, K. Bergh, Z. Duby, N. Ebrahim, T. Jankie, C. Mathews, K. Jonas

**Affiliations:** 1 Health System Research Unit, South African Medical Research Council, Cape Town, South Africa; 2 Department of Psychology, University of Cape Town, Cape Town, South Africa; 3 School of Public Health, University of Cape Town, Cape Town, South Africa; 4 Networking HIV & Aids Community of Southern Africa, Cape Town, South Africa

**Keywords:** acceptability, adolescent girls and young women (AGYW), Safe Spaces, sexual and reproductive health (SRH) services, South Africa

## Abstract

**Background and Objectives:**

Safe Spaces are community-based settings where adolescent girls and young women (AGYW) are offered biomedical, behavioral, and structural services without discrimination. We investigated the uptake and acceptability of sexual and reproductive health (SRH) and related services provided to AGYW through Safe Spaces as part of the My Journey Programme, a large-scale combination human immunodeficiency virus (HIV) prevention program implemented in South Africa.

**Methods:**

We used data from the HERStory3 study—mixed methods impact evaluation of the My Journey Programme. The quantitative component comprised a household survey of AGYW aged 15–24 years in which participants completed a self-administered electronic questionnaire, and dried blood spot specimens were used to test for HIV. We analyzed survey data from 2,608 AGYW living in the 12 subdistricts in which the intervention was implemented. A multilevel model was used to determine the association between spending time at a Safe Space in the past year and uptake of SRH services, educational enrolment, and wellbeing. A subsample of 68 survey participants took part in qualitative in-depth interviews, which were audio-recorded, transcribed, translated into English, and analyzed using thematic analysis.

**Results:**

Among participants who spent time at a Safe Space in the past year (*n* = 1,201), HIV testing (51.2%) and homework support (24.4%) were the most common services received by participants, and 81.3% were satisfied or very satisfied with the services they received, as reiterated in participants’ narratives. Participants who spent time at a Safe Space in the past year were 33% more likely to have had an HIV test in the past 6 months (95% CI: 1.06–1.67; *p* = 0.014), 69% more likely to be enrolled in an educational institution (95% CI: 1.32–2.16; *p* = 0.001), and 40% (95% CI: 1.14–1.71; *p* = 0.001) more likely to be flourishing. Interview participants also expressed appreciation for the welcoming staff at the Safe Spaces, who provided them with valuable information that increased their knowledge and uptake of SRH services.

**Conclusion:**

Given the high levels of acceptability for Safe Spaces and positive health and health-related outcomes, Safe Spaces should be included as an important component of youth-friendly SRH programs.

## Introduction

Adolescent girls and young women (AGYW) in sub-Saharan Africa face many challenges when accessing sexual and reproductive health (SRH) services, especially those living in low-income areas (1). Furthermore, their adolescent years represent a period of significant biological, psychological, and behavioral changes that make them more likely to engage in behaviors that increase their risk of exposure to sexually transmitted infections (STIs) such as human immunodeficiency virus (HIV), as well as unexpected pregnancies and early childbearing (2, 3). This is of particular concern in South Africa, where AGYW aged 15─24 years have an HIV prevalence double that of their men counterparts, and one in four adolescent girls falls pregnant before the age of 20 years (4, 5). Yet access to SRH education and services is limited and, in some cases, nonexistent (2, 3). There is a need for youth-friendly SRH services for AGYW, such as those offered through Safe Spaces, in low-income communities in South Africa.

Safe Spaces for AGYW are defined as formal or informal places where they can feel emotionally and physically safe (6). In practice, Safe Spaces are secure, private spaces where AGYW can spend time and access SRH services and social support. Safe Spaces, such as community-based girl groups, have been implemented in low-income communities in sub-Saharan African countries since 2007 to support AGYW and offer these health services (7, 8).

In sub-Saharan African countries, programs offering Safe Spaces have provided education on various topics from SRH and HIV prevention to life skills and economic strengthening (8). They have also provided SRH services, such as HIV testing, contraception, and instructions and counseling on how to discuss HIV and other STIs with partners (9). Qualitative data indicate that Safe Spaces have a positive impact on promoting self-esteem and social support, assist adolescents in developing positive life and communication skills, and improve SRH knowledge (10). Adolescent girls and young women who attend Safe Spaces report having higher expectations for education, employment, and health-related outcomes, suggesting that these spaces provide AGYW with a deeper understanding of the SRH issues they face, increasing their desire to improve their SRH and wellbeing (11).

However, multi-level barriers influence and constrain access to and use of Safe Spaces by AGYW in sub-Saharan Africa (1, 12). For instance, some AGYW have insufficient knowledge about youth-friendly health services, and access to reproductive health information is hindered by stigma related to parental consent and AGYW’s age (1, 12). Additionally, not all communities offer a Safe Space for AGYW, and there are often difficulties in finding a safe and central facility that will be easily accessible to AGYW to attend, as Safe Spaces are often moved around, or they are not well-equipped, or they may not be conducive to providing privacy to discuss sensitive topics (1, 6, 13). Thus, AGYW need information about where to access Safe Spaces, and Safe Spaces need to be safe and accessible to AGYW. This study uses mixed methods to examine AGYW’s exposure to, uptake, and acceptability of health services provided through Safe Spaces in South Africa, specifically focusing on those offered by one of the largest donor-funded HIV prevention programs—the My Journey Program.

## Methods

### Background to Safe Spaces in the My Journey Programme

The My Journey Programme has been a combined HIV prevention program for AGYW, implemented in 12 high HIV-burdened subdistricts in South Africa since 2016. The Programme aimed to lower HIV incidence, teenage pregnancy, and gender-based violence (GBV) and expand retention in school and access to economic opportunities. The Programme offered core and layered services provided by South African civil society organizations in several locations, including schools, Technical Vocational Education and Training (TVET) colleges, dedicated Safe Spaces, and mobile clinics. Core services were offered first and included enrolment and consent, an HIV risk and vulnerability assessment, and a service plan. Layered services, including biomedical, behavioral, and structural interventions, were offered based on the risk assessment findings. The layered services that were delivered through the dedicated Safe Spaces included grief support, rape prevention, substance awareness groups, HIV testing, individual and group counseling, recreational activities, homework support, provision of menstrual dignity packs for girls in need, and referral to health and health-related services. The Programme was evaluated and conducted by the Health Systems Research Unit at the South African Medical Research Council. The Programme and evaluation were both funded by the Global Fund to Fight AIDS, Tuberculosis, and Malaria through NACOSA, Beyond Zero, and the AIDS Foundation of South Africa (AFSA) (14).

### Household survey

#### Sampling and data collection

Between 24 January and 15 May 2024, a post-intervention survey was conducted in 12 intervention (where the My Journey Programme was implemented) and 12 matched comparison subdistricts, comprising rural, urban, and semi-urban settings across eight provinces in South Africa, emulating a non-randomized cluster trial. A list of the intervention subdistricts and additional information about the areas where the study was conducted are provided in the Supplementary material. The comparison subdistricts were not included in these analyses. The study consisted of four hierarchical levels of design and sampling: (1) All 12 intervention subdistricts where the My Journey Programme had been implemented were selected. Afterwards, the comparison subdistricts were selected to be equivalent in terms of demographics and HIV prevalence in 2015/16 before the intervention started (27–29); (2) Sites were purposefully selected within each intervention and comparison subdistrict by consulting the Program implementers to identify sites where the intervention was active, and Geographic Information System-based information was used to identify equivalent sites in the comparison subdistricts. All non-residential (industrial, recreational, commercial, etc.) areas were removed from the sample; (3) Dwellings were considered appropriate households if there were eligible AGYW residing there. (4) AGYW between the ages of 15 and 24 years were eligible for the study if they lived in the selected household and consented to all parts of the study. Participants who were cognitively or mentally challenged, mute or deaf, or who did not speak any of the study languages were not eligible to participate. Once informed consent was given, dried blood spot (DBS) samples were collected from participants to test for HIV, whereas socio-demographics, sexual behavior, and access to and use of SRH services were reported through a self-completed electronic survey with audio assistance. The survey was available in 10 of the 11 official South African languages, which were primarily spoken in the study areas. This study only used data from the intervention sites, as we know that Safe Spaces of a similar standard were available in these areas through the My Journey Program.

#### Measures

Multilevel logistic regression models were tested in this study. The independent variable for all the regression models was whether the participant reported spending time at a Safe Space in the past year. Safe Spaces were defined for participants as places in their community where young people can access services, interact with other young people, and participate in activities. The dependent variables for the four regression models are as follows: (1) reporting that you had an HIV test in the past 6 months; (2) reporting that you have ever used contraception methods other than condoms (such as injectables, oral contraceptives, implants or intra-uterine devices); (3) reporting that you were enrolled in school, college or university full-time; (4) and having high levels of wellbeing (flourishing). Flourishing is a composite variable that was created using a measure of high levels of emotional, psychological, and social wellbeing as defined by the Mental Health Continuum–Short Form (MHC-SF) (15, 16).

#### Data analysis

Descriptive statistics were used to report the frequencies and percentages of socio-demographic characteristics and of exposure to and uptake of health services through Safe Spaces among our sample of AGYW from the intervention sites only. Multilevel logistic regression models were used to test the relationship between spending time at a Safe Space in the past year: (1) having an HIV test in the past 6 months, (2) ever using contraception other than condoms, (3) being enrolled in an educational institution full-time, and (4) flourishing. The regression model was adjusted for appropriate covariates that included the study arm (intervention or comparison arm), age group, ever had sex, maternal orphanhood, enrollment in an educational institution (except in regression 3), where this is the main outcome, tap in house, household has a car, and ever been pregnant. The multilevel model accounted for clustering at the level of the household, the site, and the subdistrict.

## Qualitative interviews

### Sampling and data collection

In the survey consent form, participants from the intervention sites were asked whether they would be willing to be contacted by the research team and invited to participate in an interview. Willing participants were asked to enter their cell phone numbers. The research team contacted willing participants from the different intervention sites.

In-depth interviews were conducted with 68 AGYW aged 15–24 years from March to May 2024. Interviews were conducted by trained women interviewers who were fluent in the study languages, held qualifications ranging from a bachelor’s to a master’s degree, and had received study-specific training as well as training in research ethics. Both once-off and serial interviews were conducted telephonically with AGYW living in the 12-intervention subdistricts, in participants’ choice of seven official South African languages (isiZulu, isiXhosa, Afrikaans, Setswana, SeSotho, siSwati, and English). Interviews followed semi-structured discussion guides developed collaboratively with the evaluation team, funders, and implementing partners. Interview guides asked participants about their interactions and experiences with the My Journey Programme and included specific questions relating to Safe Spaces. Participants were asked whether they had attended Safe Spaces and their reasons for doing so. If they had attended Safe Spaces, they were asked to describe their experiences, any services they received, or activities they participated in there. Interviews were audio-recorded using Microsoft Teams (31).

### Data analysis

Interview audio recordings were directly translated from their original language into English transcripts. Transcripts were analyzed using a collaborative reflexive thematic analysis approach, following the six-phase framework outlined by Braun and Clarke (17). The process began with familiarization, during which the research team read and re-read transcripts while noting initial impressions and analytic memos. In the coding phase, an initial deductive codebook was developed based on key topics from the interview guide. This codebook evolved through inductive coding, as new insights and meanings emerged from the data across multiple rounds of collaborative discussion. During theme development, related codes were grouped, mapped, and refined to identify overarching patterns that captured the essence of participants’ experiences. The review and refinement phase involved iteratively revisiting coded data and memos to ensure that each theme was coherent, distinctive, and grounded in participants’ narratives. Finally, in the defining and naming stage, the research team agreed on final theme names and descriptions that best reflected the data’s underlying meaning and the study’s objectives (17, 18).

### Ethics approval

Ethics approval for the study was granted by the Human Research Ethics Committee (HREC) at the South African Medical Research Council (EC027-8/2023). An informed consent process was conducted with participants for both the electronic survey and the telephonic interviews. For the electronic survey, participants gave written (digital) consent, and signed consent forms are stored on a password-protected computer. For the telephonic interviews, verbal consent was audio-recorded and stored on a password-protected computer.

For participants under 18 years of age, parental/guardian consent was obtained before participants were asked to provide their own consent. Participants received a R200 (US$11) reimbursement in the form of a voucher for groceries, cell phone data, or electricity for study participation and a R50 (US$2.84) reimbursement for the household.

## Results

### Quantitative results

Across study arms, 37,714 dwellings were visited, 22,263 households were screened, 5,150 AGYW were invited, and 5,025 participated (97.5% response rate). Of the 5,025 survey participants, 4,932 (98.2%) had available DBS data (sample in Tables 1–3). Among participants with available DBS data, 2,604 (52.8%) were from the intervention arm and were included in these analyses.

**Table 1:** Participant characteristics and use of Safe Spaces among participants in the intervention arm, stratified by age group (*n* = 2,604).

Variable	Age group				Total	
	15–19 years		20–24 years			
	*N*	%	*N*	%	*N*	%
Tap in-house	700	46.9	535	48.1	1,235	47.4
The household has a car	357	23.9	239	21.5	596	22.9
Enrolled in an educational institution (school, TVET college, university) full-time	1,137	76.3	468	42	1,605	61.6
Mother has died	170	11.4	226	20.3	396	15.2
Mother alive	1,305	87.5	876	78.7	2,181	83.8
Ever had sex	731	49	985	88.5	1716	65.9
Ever been pregnant	252	16.9	646	58	898	34.5
Ever used contraception	463	31.1	740	66.5	1,203	46.2
Had an HIV test in the past six months	682	45.7	819	73.6	1,501	57.6
HIV status						
DBS-confirmed HIV positive	93	6.2	181	16.3	274	10.5
Knew of an organization providing Safe Spaces for AGYW in their community	676	45.3	474	42.6	1,150	44.2
Spent time at a Safe Space in the past year	711	47.7	490	44	1,201	46.1

**Table 2:** Services received at Safe Spaces in participants’ communities among participants in the intervention arm who reported spending time at a Safe Space in the past year, stratified by age group (*n* = 1,201).

Variable	Age group				Total	
	15─19		20─24			
	*N*	%	*N*	%	*N*	%
Received an HIV test	321	45.1	294	60.0	615	51.2
Received contraception	62	8.7	95	19.4	157	13.1
Received pre-exposure prophylaxis (PrEP)	33	4.6	53	10.8	86	7.2
Received antiretroviral therapy (ART)	29	4.1	11	2.2	40	3.3
Received condoms	81	11.4	123	25.1	204	17.0
Homework support	209	29.4	84	17.1	293	24.4
Supporting getting a job	21	3.0	68	13.9	89	7.4
Adherence support and clubs	2	0.3	7	1.4	9	0.7
Received help from a social worker	37	5.2	24	4.9	61	5.1
Received counseling to help with distress or grief	19	2.7	20	4.1	39	3.2
Participated in a self-defense class	18	2.5	9	1.8	27	2.2
Participated in a parenting class	6	0.8	10	2.0	16	1.3
Connected to Wi-Fi or the internet	73	10.3	45	9.2	118	9.8
Joined a music, game, or fun activity	105	14.8	41	8.4	146	12.2
Received services from a mobile clinic at the Safe Space	30	4.2	29	5.9	59	4.9
Sports activity	141	19.8	66	13.5	207	17.2

**Table 3:** Acceptability of Safe Spaces in the community among participants in the intervention arm who reported spending time at a Safe Space in their community in the past year, stratified by age group (*n* = 1,201).

Variables	Age group				Total	
	15–19		20–24			
	*N*	%	*N*	%	*N*	%
How comfortable are Safe Spaces for AGYW?						
Not comfortable	32	4.5	35	7.1	67	5.6
Unsure	133	18.7	76	15.5	209	17.4
Comfortable	497	69.9	352	71.8	849	70.7
Prefer not to answer	49	6.9	27	5.5	76	6.3
How satisfied were AGYW with the services provided at Safe Spaces?						
Very dissatisfied	10	1.4	8	1.6	18	1.5
Dissatisfied	9	1.3	4	0.8	13	1.1
Neither satisfied nor dissatisfied	30	4.2	15	3.1	45	3.7
Satisfied	216	30.4	138	28.2	354	29.5
Very satisfied	344	48.4	278	56.7	622	51.8
Prefer not to answer	102	14.3	47	9.6	149	12.4

Participant characteristics are described in Table 1, stratified by age group. The percentage of participants in the 15–19 age group was 57.3%. Across age groups, 57.6% of participants had HIV tests in the past 6 months, and 10.5% of participants were DBS-confirmed HIV positive. Overall, 65.9% of participants had ever had sex, and 46.2% reported ever using contraceptives. Almost two-thirds of participants (61.6%) reported being enrolled in an educational institution. In terms of wellbeing, 65% of participants reported that they were flourishing, meaning that they had high levels of wellbeing. Regarding Safe Spaces, 44.2% of participants had heard of an organization providing Safe Spaces for AGYW in their community, and 46.1% had spent time at a Safe Space in the past year.

The services received by participants at the Safe Spaces are described in Table 2 among participants who reported spending time at a Safe Space in the past year, stratified by age group. Across age groups, 51.2% of participants received an HIV test at the Safe Space, 13.1% received contraception, and 17% received condoms. Furthermore, 24.4% of participants received homework support. In terms of activities, 12.2% of participants engaged in a music, game, or fun activity, and 17.2% took part in a sports activity.

Participants’ perspectives on the acceptability of Safe Spaces are presented in Table 3 among participants who reported spending time at a Safe Space in the past year. Across age groups, 70.7% of participants reported feeling comfortable at the Safe Spaces, and 81.3% were satisfied or very satisfied with the services they received there.

For the multilevel logistic regression analysis, each regression was adjusted for the following potential covariates: study arm, age group, ever had sex, maternal orphanhood, educational enrolment, tap in house, household has a car, and ever been pregnant. Regarding uptake of SRH services, participants who spent time at a Safe Space in the past year were 33% (OR = 1.33; 95% CI: 1.06–1.67; *p* = 0.014) more likely to have an HIV test in the past 6 months compared to participants who had not spent time at a Safe Space in the past year. However, participants who spent time at a Safe Space in the past year were neither more nor less likely to have ever used contraceptives compared to those who had not spent time at a Safe Space in the past year (OR = 1.14; 95% CI: 0.89–1.46; *p* = 0.317). Regarding educational outcomes, participants who spent time at a Safe Space in the past year were 69% (OR = 1.69; 95% CI: 1.32–2.16; *p* < 0.001) more likely to be enrolled in an educational institution full-time compared to participants who had not spent time at a Safe Space in the past year. Lastly, participants who had spent time at a Safe Space in the past year were 40% (OR = 1.40; 95% CI: 1.14–1.71; *p* = 0.001) more likely to be flourishing compared to participants who had not spent time at a Safe Space in the past year.

## Qualitative results

### Acceptability of Safe Spaces

#### AGYW’S perceptions of Safe Spaces staff

Generally, AGYW’s narratives suggested a high level of acceptability of Safe Spaces as a place for accessing health and health-related services. The high levels of acceptability reported by participants were attributable to the welcoming demeanor of the staff and the quality of services received. Participants explained that staff at the Safe Spaces were friendly, helpful, encouraging, and approachable, and that they provided excellent treatment.

*I have (attended a Safe Space). It was very welcoming, very good… very nice people… welcoming with warm hearts…. The people give you full information on whatever you want to know, they explain everything in detail… very welcoming (AGYW 18-24 years, Gauteng).*

Participants’ narratives suggested that AGYW perceived the staff at Safe Spaces as less judgmental and harsh than the staff at government health facilities. In the quote below, the participant indicated that she prefers to access services at Safe Spaces rather than at community clinics.

*Even the health workers there (at the Safe Space). They are not so harsh, everything is nice… I prefer the Safe Space with the health workers and social workers rather than the clinic health workers. They very much want to help, it’s nice… without judging you, without being rude (AGYW 18-24 years, Gauteng).*

Another positive view expressed by AGYW was that participants perceive Safe Spaces as youth-friendly services with highly trained staff.

*I had advised people to go there (Safe Space). I applaud them for being nice people… for giving information, being dedicated to their work, and knowing how to speak to a person, and also helping a person. So, my experience is good. I would recommend and also applaud them for the treatment they gave us…(AGYW 18-24 years, Gauteng).*

The participant below described how she received friendly treatment, support, and advice when she took an HIV test for the first time at a Safe Space. She further explained that she was able to share if something is bothering her.

*I did get (HIV testing) there (Safe Space)… people from there they are friendly people, I mean…they know how to communicate with people in a good way, even when you’re no longer feeling well, you can tell them that no, there is something like this and that… so that they can do what? …advise you may be to motivate you… I went there for the first time for testing for HIV. (AGYW 1824 years, Free State).*

Participants further explained that at Safe Spaces, they received SRH services and information without being judged and were provided services by people who are passionate about their work. Furthermore, the participant quoted below explained that she liked that the service she received was provided by someone her age, making the experience more relatable.

*Receiving the HIV testing and information (at the Safe Space), it was so easy because the person who was giving us the help there was someone in our age group. So, we could relate on a lot of things, and they knew how to ask questions, advise, and recommend some things…. Also, receiving services… it is a complete bonus because you’re helped… So, my experience was very good (AGYW 18-24 years, Gauteng).*

The openness of Safe Spaces staff was also a positive attribute for AGYW, as staff engaged openly and did not hide things. Staff raised points they perceived as important to participants; consequently, participants felt happy and realized the importance of the things discussed at Safe Spaces for adolescents.

*(I did go to the Programme Safe Space). It was good…they are open, and you should also be open and tell them this… I was happy; I realized that I needed those things as a girl child (AGYW 18-24 years, Northwest).*

#### AGYW’S perceptions of the Safe Space environment

Another positive aspect of Safe Spaces was the way in which they provided an opportunity for AGYW to get the help they needed, while also maintaining privacy and confidentiality. Participants who spent time at the Safe Space had positive views on the Safe Space environments, suggesting Safe Spaces offered an enabling environment to be themselves and feel free, while also receiving SRH services such as HIV testing.

*(Safe Space) It is where there are services; if you do not feel good, they can help you… It is a nice space; the people there are good people with good hearts. When you are there, you feel at ease (AGYW 15-17 years, Free State)*

Research participants’ narratives demonstrated a positive view of Safe Spaces’ privacy, confidentiality, and safety. One participant explained that, despite her initial concerns, she was reassured and realized that the things she shared at the Safe Space would be kept private.

*When I attended Safe Space, I was scared because I did not know if I could speak about something that was bothering me, if they would not share it with others but on my first day I saw that here is the right place for someone to share what is bothering them (AGYW 18-24 years, Eastern Cape).*

#### AGYW’S perceptions of services at Safe Spaces

The counseling services provided at the Safe Space helped to make participants feel safe and comfortable, helping them to talk about difficult topics. One participant describes how she was alone when she went for HIV testing at the Safe Space, and that she was scared about the results, but that she received the support and counseling that she needed.

*I did (attend a Safe Space). I received contraceptives… I got it alone… I did it before I got HIV results… So, I arrived there scared, and then they did counseling first (AGYW 18-24 years, Free State).*

The services provided at Safe Spaces created an opportunity for AGYW to learn and talk freely about issues affecting girls that parents do not like to discuss with them, such as rape and sexual assault. Furthermore, participants felt that the way these services were provided also ensured that the information they shared would be kept private and confidential.

*My aim… was to learn about things happening to us girls that our parents could not notice. If you do not have anyone that you can talk to at home, you also get a Safe Space there… every Thursday, there is a Safe Space for girls where you can share your story, maybe you were sexually assaulted, you can speak there. You can also write a letter if you are scared to speak with others around you, meaning that there is also privacy within us. So, there you can share your story or something that has happened to you or something that you cannot share on your own with your family or friends, but we can at the Safe Space (AGYW 18-24 years, Eastern Cape).*

### Motivations for attending Safe Spaces

AGYW described a range of services offered at Safe Spaces, including SRH education; a platform to communicate, as there is a lack of communication between AGYW and their families; social support from peers, education and career support, and economic support, which served as a motivation for AGYW to attend Safe Spaces.

Some participants said they first attended a Safe Space out of curiosity about the program and a desire to find out more about the services offered.

*(I joined the program because) I wanted to learn things there… I wanted to understand issues around HIV infection, and the importance of pregnancy… and contraceptives, so that one does not become pregnant (Dihlabeng, Thabo Mofutsanyana, AGYW 18-24 years).*

Lack of parent-adolescent sexuality communication, and the challenges AGYW experience in communicating with families about romantic relationships, encouraged AGYW to attend Safe Spaces. The participant quote below indicates that Safe Spaces have the potential to offer AGYW a space to share things they want to talk about which they are unable to discuss with their families, and that AGYW are attracted to Safe Spaces as a place for them to share and get advice.

*What made me go to Safe Space is that I am not able to talk with my family. The person I can talk with is my big sister, but she is not always around. I found out that at the Program I can vent and get advice, you see, so I thought, let me go there because there are things you cannot talk to family, things that have to do with a partner (AGYW 18-24 years, Eastern Cape).*

Another positive aspect of Safe Spaces described by participants related to academic and employment support. The provision of career support and internet access provided at the Safe Spaces attracted AGYW to Safe Spaces. The participants further explained that they were motivated to visit a place that provides support in a friendly, welcoming manner.

*Even with school-related matters and job applications, if you have no data, they help you (at the Safe Space), everything is for free…Then, after a while, they explained the importance of using contraceptives, and it made sense to me, so I applied and started, even doing a CV because I needed it… I saw it necessary for me to go to a place (Safe Space) where I know that help is guaranteed, and help is offered in a gentle way without aggression and being shouted at (AGYW 18-24 years, Free State).*

In addition to career and educational support, and health services, an additional aspect that attracted AGYW to attend Safe Spaces was the provision of food. The accessibility and acceptability of Safe Spaces were increased when they were easy to get to, when transport was available, and when the staff were friendly. *It was easy (to attend the Safe Space), it was nearby… at the nearby one they were also welcoming and I knew that I could get services of health and of social, and with help with school, I’ll go there because it’s near. And then… meals… they were also offering food… when it’s far, they give us transport, and they offer us food as well. And then, the people are not rude, they are nice (AGYW 18-24 years, Gauteng).*

The provision of counseling and psychosocial support also motivated AGYW to attend and use the services at Safe Spaces. One participant expressed that she realized that she needed the services provided at Safe Spaces, particularly counseling services, due to anger, and thus participated in individual counseling *(The services at Safe Space) they were good… I could see that I needed counseling, you can also see that I have a lot of anger, I have a lot of anger…I did individual counseling… I used to go there on.*

*Mondays and Fridays only…It happens like every week (AGYW 18–24 years, Northwest).*

Another service that motivated AGYW to attend Safe Spaces was to access HIV testing. For some of the participants, this was the first time being tested for HIV.

*What made me… go there… I went there (Safe Spaces) for the first time for testing for HIV (AGYW 18–24 years, Free State).*

### AGYW perceived the benefits of attending Safe Spaces

Increased psychological wellbeing was one of the benefits of Safe Spaces identified by participants, who reported being happy at Safe Spaces. A positive aspect of Safe Spaces described by participants related to psychosocial support, and the way in which they provided an opportunity to connect with peers and staff. Participants who spent time at the Safe Space had positive attitudes toward it because it allowed them to meet new people. At the Safe Space, participants could spend time together, creating a sisterhood and connecting with peers.

*We come together, and then it becomes one big sisterhood program (AGYW 18-24, Gauteng).*

In addition to receiving psychosocial support, AGYW reported improved skills in being able to talk about their problems and challenges, thus improving access to support.

*In that program, they could tell you what you may encounter in life and when you have problems, who to encounter… I benefited from it…. You could talk to them, and they would not give up on you. You could talk about what happened to you until you are fine and until they get help for you to ensure that you have overcome the problem (AGYW 18–24 years, Free State).*

In addition to receiving services, gaining SRH information through education provided at the Safe Spaces was also a benefit described by participants:

*The information and services I got from Safe Space and the program regarding those (SRH) topics – I am fulfilled… I feel very knowledgeable about it (AGYW 18–24 years, Gauteng).*

A participant explained that different services provided at Safe Spaces, such as the counseling she attended, assistance with job hunting processes, and business skills training, were available. AGYW were also assisted with tertiary education application forms and therefore these services motivated AGYW to attend Safe Spaces.

*At Safe Space I was attending for counseling, and there was career employment… if there is a work opportunity they will come and give out the information and there was also skills training… There was a training that we did… Another training is going to take part that is about Business management… we were told there will be other trainings for unemployed people to improve their skills and also help to look for schools for the ones who have taken gap year so they will be given application forms (AGYW 18-24 years, Eastern Cape).*

Support with CV creation and assistance with job and education applications at Safe Spaces was seen as a key benefit by participants. Participants described how they received career support, such as help writing a CV and applying for jobs at no cost.

*I received it, and I am still receiving them even now because they (staff at Safe Space) help us to do a CV, they even tell us if there are posts available, they call us to come and apply, even when the institutions of higher education open, they help us apply even for NSFAS (AGYW 18–24 years, Free State).*

Safe Spaces enabled AGYW to assist and support each other with studying and homework support, leading to improved academic performance. Participants demonstrated that attending Safe Spaces, where people who have completed their undergraduate and postgraduate degrees are present, helps AGYW, as they can seek advice from people with higher education. This was associated with positive academic performance, suggesting a need for Safe Spaces.

*We assist each other with reading and writing homework. I was a part of it before and after I finished school… I am finished with grades, and am in my first year now… I think it does help because there are people who have obtained degrees and honors whom I can ask for advice on how I can do something… I benefit… secondly, my marks improve, and I can find certain ways of how to study and manage my time when I study (AGYW 18–24 years, Gauteng).*

Gender-based violence (GBV) and self-defence services were also provided at Safe Spaces. Participants who participated in self-defence classes indicated that these classes were empowering and had a positive impact on their lives.

*I participated in it (GBV support/self-defence program held at the Safe Space)… You can protect yourself, as people like to abuse others, and they talk about such abuse that is happening…. They said someone may just attack and injure you for no apparent reason…. and that we should stop being on the streets at night because it is not safe, there are a lot of things happening outside….It positively changed my life (AGYW 18–24 years, Free State).*

## Discussion

This study aimed to examine AGYW’s exposure to and uptake and acceptability of health services provided through My Journey Safe Spaces in South Africa using data from a household survey and in-depth interviews from communities in which the My Journey Programme was implemented. The quantitative survey data suggest that exposure to Safe Spaces was fairly high, as almost half of the survey participants had spent time at a Safe Space in the past year (46%). Furthermore, services were highly acceptable to participants, with 81% of participants reporting that they were satisfied or very satisfied with the services that they received. This finding was reinforced by the qualitative data in which participants described how they found Safe Spaces welcoming and with friendly staff who supported them to adhere to the services they received. HIV testing and homework support were the most common services received by participants, and AGYW who attended a Safe Space in the past year were more likely to have had an HIV test in the past 6 months and be enrolled in an educational institution, such as a school, TVET college, or university full-time. AGYW who had spent time at a Safe Space in the past year were also more likely to have high levels of wellbeing. These findings are supported by participant narratives describing how attendance at Safe Spaces encouraged HIV testing, school enrollment and mental health, by providing SRH education and services, homework and career support and counseling services, motivating participants to attend the Safe Spaces. Benefits of attending a Safe Space included increased academic performance, knowledge about GBV, and the ability to speak more openly about difficult topics.

Regarding the acceptability of Safe Spaces, our study suggests that Safe Spaces were perceived as an enabling environment for AGYW to access services. Most participants who used them reported that Safe Spaces were comfortable places for AGYW to spend time (71%), as staff were friendly and provided high-quality care. Participants specifically appreciated staff openness and relatability, which allowed them to engage freely and share their challenges. Participants explained that there was greater privacy and less judgment and harshness when receiving services at the Safe Space compared to public health facilities. A study conducted in South Africa’s Eastern Cape province also found that Safe Spaces were acceptable: AGYW reported high satisfaction with the SRH services because the staff were welcoming and non-judgmental (19). AGYW often report a lack of privacy when accessing SRH services in community clinics, even when these are youth-friendly clinics (20). An earlier mixed methods process evaluation of the My Journey Program conducted in six of the same districts as our study showed that Safe Spaces overcame concerns about privacy by allowing AGYW to learn to engage in discussions about SRH and to access services in a convenient, accommodating environment.

In qualitative interviews, participants expressed various motivations for attending Safe Spaces, including the desire to address information gaps caused by a lack of sexuality communication in families. In many communities in sub-Saharan Africa, adolescents are perceived as too young to discuss romantic and sexual relationships as well as other topics relating to SRH, especially by parents, leading to a lack of sexuality communication between adolescents and their families, mostly influenced by religious and cultural beliefs (21, 22). Our study reinforces this evidence, as AGYW reported being motivated to attend Safe Spaces because they could speak freely about SRH-related topics there, especially for those from households where communication between AGYW and their families is limited. As shown in our findings and previous research, open SRH communication at Safe Spaces was a key motivator for AGYW to attend Safe Spaces (23). While a scoping review of Safe Spaces in high-income countries (Finland, Spain, and the Netherlands) has already shown that Safe Spaces enhance SRH knowledge for improved sexual decision making (22), our study supports the growing body of evidence from low and middle-income countries that Safe Spaces can effectively offer urgently needed accurate sources of SRH information and support to AGYW for safe and healthy sexual decision-making (21).

There is currently a high unmet need for contraception among AGYW in South Africa (30). Accessing SRH services, such as receiving and utilizing pregnancy prevention methods and testing for HIV, was a key motivation for and benefit of attending Safe Spaces. Our qualitative findings show that AGYW participants were interested in learning more about contraception, and this motivated them to attend Safe Spaces. However, the quantitative findings showed that only 13% of participants reported receiving contraception from a Safe Space, and attending a Safe Space in the past year was not associated with ever using contraception. Evidence from high-income countries shows that Safe Spaces encourage healthy behaviors such as increased use of contraceptives (22), and it is important to find out why My Journey Safe Space attendance did not increase contraceptive use. While our quantitative findings revealed that participants who spent time at a Safe Space in the past year were not more or less likely to have ever used contraceptives, they were more likely to have had an HIV test in the past 6 months, suggesting that there are other, more complex sociocultural barriers to contraceptive uptake (24). The findings align with Bhushan et al.’s (9) study, which was conducted in South Africa and demonstrates that AGYW who were part of the Safe Spaces programs were more likely to receive HIV testing.

Career and educational support were another factor that attracted AGYW to Safe Spaces. Qualitative findings indicated that AGYW were attracted to Safe Spaces by the provision of employment support at Safe Spaces. Participants explained that they were assisted with job-seeking processes by being notified of job opportunities, receiving Business Management training, and being helped with CV writing. Findings from the process evaluation of the My Journey Program showed that Safe Spaces offered internet services that AGYW could utilize for job applications (6). This suggests a need to maintain employment support services at Safe Spaces to ensure they have an internet connection. Educational benefits of Safe Spaces were another key finding: participants who attended a Safe Space in the past year were more likely to be enrolled full-time in an educational institution, and qualitative narratives described how services at the Safe Space helped improve academic performance. These services included receiving peer support for homework and studying, as well as assistance from individuals with higher education, thereby encouraging and enhancing school attendance. Another study found that participants in the DREAMS intervention in Kenya reported that completing tertiary school was important to them (11). This shows how Safe Spaces go beyond SRH services to provide essential educational services to AGYW, which they both need and want.

Psychosocial support, mental health, and wellbeing were also key benefits of Safe Spaces. The qualitative findings revealed that participants often attended Safe Spaces for recreational activities, which allowed them to develop connections with their peers and support each other. Furthermore, these findings align with the study by Mathews et al. (6) on My Journey Safe Spaces, which revealed that AGYW are often attracted to Safe Spaces by the recreational activities offered, but once they can relax and connect with their peers. This might benefit our participants’ psychosocial wellbeing, as the quantitative findings show that participants who spent time at a Safe Space in the past year were more likely to flourish. The counseling services provided at Safe Spaces also motivated AGYW to attend. One participant explained that she needed counseling as she realized that she had a lot of anger, and Safe Spaces came to her rescue. In support of this, a qualitative study conducted in the Eastern Cape, South Africa, found that counseling services at Safe Spaces significantly improved participants’ wellbeing (19). This underscores the value of counseling services for AGYW; therefore, they should be made available in all youth services centers. Furthermore, gaining knowledge about GBV and self-defense services was among the benefits of attending the My Journey Safe Spaces. Although only 2% of participants reported attending a self-defense class in our survey, those who did said that it had a positive impact on their lives. Furthermore, the qualitative narratives describe in detail how Safe Spaces increased AGYW’s knowledge and awareness of GBV. This is a critical issue in South Africa, as approximately 8% of AGYW in South Africa report rape from intimate partners and non-intimate partners (25).

## Strengths and limitations

This study contributes to the evidence base on the value of Safe Spaces for AGYW, particularly given the limited research on the acceptability of Safe Spaces, and supports the implementation of Safe Spaces for AGYW. Future studies should focus on awareness of Safe Spaces, especially in remote communities, and barriers to accessing and using Safe Spaces.

Limitations include that the study was cross-sectional and we cannot be sure whether Safe Spaces were associated with greater HIV testing, greater school enrollment and a greater sense of flourishing, or whether AGYW who already had been tested, who were already enrolled in school or were already flourishing found it easier to access them subsequently. Another limitation is that we did not ask participants how many times they visited the Safe Spaces. However, we asked participants how recently they attended the Safe Space (in the past year), which would also affect the relationship between attendance and health outcomes. While our measure of exposure to Safe Spaces used in statistical analyses does not include participants’ reasons for attending a Safe Space, we know from other variables described in Table 2 that most participants received SRH and educational services at the Safe Space. The prevalence of some services listed in Table 2 was very low, and we speculate that participants did not scroll to the bottom of the list on the tablet when answering the question. Both the electronic survey and qualitative interviews may be subject to social desirability bias, although steps were taken to minimize bias, including a comprehensive informed consent process. Lastly, participants were provided with incentives after completing the interview or survey to thank them for their time, and they were informed of this during the consent process. This might have encouraged them to provide more positive responses.

## Implications and conclusions

It is evident that services at Safe Spaces were acceptable to AGYW beneficiaries of the My Journey Program, and that these offered motivated them to attend and utilize Safe Spaces. While discussions about SRH, access to contraceptives, and employment support were reported to motivate participants to attend Safe Spaces, uptake of the actual SRH services was mixed, with attendees being more likely to have received HIV testing, but neither more nor less likely to have ever received contraception. Future research is needed to understand why Safe Spaces did not improve uptake of contraception, and Safe Spaces should ensure that they encourage uptake of SRH services even if that is not the main driver for AGYW to attend Safe Spaces.

Programs need to ensure that the Safe Spaces they provide are safe and convenient to access for AGYW, as some participants had heard about Safe Spaces but never attended. Safety and convenience may also be the reasons that participants did not take SRH products home. Furthermore, AGYW should not need to travel long distances to receive SRH services such as contraception and educational support. Hence, identifying appropriate locations for Safe Spaces is essential, and including AGYW in identifying these locations and developing safe travel plans could ensure that AGYW feel comfortable accessing them. Evidence from a school-based intervention in South Africa has shown that Safe Spaces at school are highly acceptable to participants. Yet, there remains a need for Safe Spaces in the community for older and non-school-going AGYW (26). Finally, our findings highlight a need for academic and employment support and counseling services for AGYW, which are unlikely to be offered in other public schools or health facilities.

## Data Availability

The original contributions presented in the study are included in the article/Supplementary material, further inquiries can be directed to the corresponding author.
