# Supplementary material for: Uptake and acceptability of health services for adolescent girls and young women provided through Safe Spaces in South Africa
Source: Front Public Health. 2026 Mar 30;14:1797269. doi: 10.3389/fpubh.2026.1797269 (PMC13071039; doi:10.3389/fpubh.2026.1797269)
Supplement: Supplementary file 1 [file Table_1.DOCX]

| **Provinces** | **District** | **Intervention** | |
| --- | --- | --- | --- |
| **KwaZulu-Natal** |  | **Subdistrict** | **Site** |
|  | Zululand | Abaqulusi | Coronation,Nkongolwane |
|  |  |  | Vryheid |
|  | King Cetshwayo | City of UMhlathuze | Empangeni |
|  |  |  | Esikhaweni H |
| **Mpumalanga** | Ehlanzeni | Mbombela | Phola |
|  |  |  | Matsulu |
|  | Gert Sibande | Govan Mbeki | Embalenhle |
|  |  |  | Embalenhle |
| **Eastern Cape** | Nelson Mandela Bay Metro | Nelson Mandela C | Malabar |
|  | Oliver Tambo | Nyandeni | Maurbeni |
|  |  |  | Nkanga |
| **Free State** | Thabo Mofutsanyana | Dihlabeng | Fateng Tse Ntsho |
|  |  |  | Kgubestwana/Mashaeng |
|  |  | Setsoto | Moemaneng |
|  |  |  | Meqheleng |
| **Limpopo** | Greater Sekhukhune | Fetakgomo Tubatse | Mpahanama |
|  |  |  | Praktesseer |
| **Gauteng** | Tshwane | Tshwane 1 | Soshanguve** |
|  |  |  | Garankuwe |
| **North West** | Bojanala | Rustenburg | Boitekong |
|  |  |  | Boitekong |
| **Western Cape** | City of Cape Town | Kilpfontein | Athlone |
|  |  |  | Hanover Park |
| **Total** |  | **12** | **24** |

Supplementary Table 1: List of subdistricts for My Journey Programme intervention arm
